# Behavioral and emotional symptoms and quality of life in a national sample of individuals with CLN3 Batten disease

**DOI:** 10.1186/s13023-026-04436-w

**Published:** 2026-06-11

**Authors:** Beate Oerbeck, Ingrid B. Helland, Heather R. Adams, Kristin Romvig Overgaard

**Affiliations:** 1 Division of Mental Health and Addiction, Oslo University Hospital, Oslo, Norway; 2 Division of Pediatric and Adolescent Medicine, Oslo University Hospital, Oslo, Norway; 3 Departments of Neurology & Pediatrics, University of Rochester Medical Center, Rochester, New York USA; 4 Institute of Clinical Medicine, University of Oslo, Oslo, Norway

**Keywords:** Juvenile neuronal ceroid lipofuscinosis, Spielmeyer-Vogt disease, CBCL, ABCL

## Abstract

**Background:**

CLN3 Batten disease, also known as Juvenile Neuronal Ceroid Lipofuscinosis, is a childhood-onset neurodegenerative disorder caused by mutations in the CLN3 gene, frequently accompanied by emotional and behavioral symptoms. Although these symptoms and a reduced quality of life have been previously reported in individuals with CLN3 disease and their parents, no published comparisons with siblings exist. Furthermore, age and sex differences require further study. In a nationally representative sample (n = 20, age 7-29 years), the present study applied the ASEBA Behavior Checklists, the Inventory of Life Quality to examine the prevalence of emotional and behavioral problems and quality of life in affected individuals and sibling controls. Additionally, we assessed parental quality of life using the Satisfaction With Life Scale.

**Results:**

We found that individuals with CLN3 disease scored in the borderline clinical range for the ASEBA total problem scores (mean 63.1, SD 11.6), whereas siblings fell within the normal variation range (mean 46.4, SD 11.4). A severely reduced quality of life was reported for both affected individuals and their parents, while siblings scored within the normal range. No significant sex differences were found. Despite facing significant challenges, families also reported strengths in their offspring: grit, determination and a sense of humor in affected individuals, and empathy and dutifulness in sibling controls.

**Conclusions:**

This study underscores the significant emotional and behavioral challenges faced by individuals with CLN3 Batten disease, which affect the quality of life for them and their parents. These findings emphasize the need for targeted interventions for both the patients and their families.

## Background

CLN3 Batten disease, also known as Juvenile Neuronal Ceroid Lipofuscinosis, is a rare and debilitating pediatric neurodegenerative disorder caused by pathogenic variants in the CLN3 gene. The disorder is characterized by the accumulation of lysosomal storage material in neurons, resulting in progressive neurological decline, visual impairment, and severe cognitive and motor dysfunction [1]. Clinically, it is well-known that individuals with CLN3 disease frequently develop emotional and behavioral symptoms, including anxiety, depression, aggression, and psychotic symptoms. Despite this clinical knowledge, there remains a significant paucity of research quantifying these symptoms in affected individuals.

To date, two studies, carried out in Finland [2] and the USA [3] have utilized standardized parent-proxy questionnaires to evaluate the frequency and severity of emotional and behavioral symptoms. In both studies, the Child Behavior Checklist (CBCL) was available for 24 individuals, and the results from both corroborated previous clinical observations, with the CBCL total problem score falling within the borderline clinical range (T-scores 60–64). The Finnish study reported that social, thought, and attention problems were particularly prominent. They also identified sex differences in symptom reporting, with females exhibiting more anxiety and depressive symptoms and attention problems. In contrast, the US study did not observe significant sex differences in symptom levels and called for further research on the topic. The CBCL subscales with the highest frequencies of borderline or clinical elevations were “thought”, “social”, “attention” and “aggressive” behavior problems. Additionally, the US study found that chronological age did not significantly correlate with the CBCL total problem score. However, in a later larger study with children and adults, also using the CBCL, behavioral problems increased through the middle teen years and then decreased, showing an inversed U-curve [4].

A subsequent large study noted that female individuals with CLN3 disease had lower functional capability and experienced earlier loss of independent physical function compared to males [5]. Using the Paediatric Quality of Life Inventory (*n* = 49), the study also found that females had a lower physical quality of life (QoL), than males. In addition, the slope of disease progression was more rapid for females, with symptom onset one year later, and death one year earlier than their male counterparts. This study emphasized the importance of future research into sex differences in CLN3 disease, which could further illuminate the biological mechanisms underlying disease progression and potentially guide the development of targeted therapies.

Recently, maladaptive behaviors were found to be common in individuals with CLN3 disease (11/17, 65%) using the Vineland Adaptive Behavior Scales, Third edition (Vineland-3), but sex differences were not explored [6]. Furthermore, the progression of CLN3 disease and its impact on family life were examined through qualitative interviews with parents (*n* = 39) of affected individuals [1]. The parents reported a substantial number of symptoms in their affected children, including anxiety (70%), depression (40%), behavioral problems (74%) and obsessive-compulsive behaviors (65%), though sex differences were not explored. In addition, the study highlighted the significant financial and interpersonal strain experienced by families, with many reporting altered work commitments and marital challenges related to coping with the disease. Additionally, siblings of chronically ill children often face emotional and psychological strains that affect their own QoL [7, 8]. However, findings are not unambiguous, as QoL was not reduced in siblings (*n* = 174) of a diverse group of chronically ill children [9]. Nevertheless, for siblings of children with life-limiting conditions, both parental and self-reported QoL have been found to be reduced [10].

There is a notable research gap concerning emotional and behavioral problems and QoL in individuals with CLN3 Batten disease, as well as QoL in their families. Additionally, no previous studies have employed sibling controls to account for genetic predispositions to emotional and behavioral disorders, to enhance the validity of the findings.

## Methods

### Aims

This case-control study aims to expand upon this limited body of research by examining the occurrence rates of emotional and behavioral symptoms in a national cohort of individuals with CLN3 disease, alongside quality-of-life assessments for them and their families. We also investigated potential age and sex differences. Based on the existing literature, we hypothesized high occurrence rates of emotional and behavioral symptoms and a reduced quality of life in affected individuals compared to sibling controls, as well as diminished quality of life for parents. While we did not have specific hypotheses regarding sex differences in symptoms of CLN3 disease, we did hypothesize a decrease in quality of life with increasing age, in affected individuals.

### Participants

Participants eligible for inclusion were parents of all living individuals in Norway diagnosed with CLN3 disease through molecular genetic testing at least one year before study start, with no other exclusion criteria. Families of 25 affected individuals were approached and 20 of them participated (80%) including one pair of siblings. Most participants (18/20) carried the classical 1 kb CLN3 deletion; of these, 14 were homozygous for the deletion and four were heterozygous. The remaining two participants carried other pathogenic CLN3 variants.

The parents completed information about the affected individuals, themselves, and if possible one sibling, preferably of the same sex and nearest in age as a comparison group [11]. While most parents discussed the child questionnaires among themselves, mothers generally completed them.

### Procedures for recruitment

The parents of children with CLN3 disease were recruited from the Norwegian NCL user organization (https://www.nncl.no/), which includes all but two of the known diagnosed cases. Members were contacted through the project’s user representative and invited to participate in the study, with consent forms provided. Upon receiving the signed consent forms, investigators sent questionnaires to the participating families, which were then returned to the researchers for analysis.

### Measures:

#### Background sociodemographic questionnaire

The parents informed on the sex, age at inclusion, age of CLN3 disease symptom start and whether the child lived at home or in care. Furthermore, parents reported their own age and educational level, whether they worked full time or not, and age and sex of the participating, unaffected sibling.

#### Information from medical records

All individuals with CLN3 disease are offered annual evaluations at the Oslo university hospital by the same pediatric neurologist (Author IH) who provided medical record information for evaluating participants’ CLN3 disease symptoms.

#### CLN3 disease symptom evaluation

The Hamburg Kohlschütter-scale was used to assess symptoms of CLN3 disease in affected individuals [12]. The scale includes five questions about the presence of seizures, and the functioning of vision, motor- language- and intellectual abilities. Each question is rated on a four-point scale (0–3) where 3 points represent an appropriate age score and 0 points indicate no residual function, with a total score calculated (range 0–15). All assessments were conducted using the Norwegian version of the scale [13] by the third author (IH), who has significant experience in clinical assessment of affected individuals.

#### Emotional and behavioral problems

Parents rated the prevalence of problems in affected individuals and sibling controls with the age appropriate Achenbach System of Empirically Based Assessment (ASEBA) questionnaires; The Child Behavior Checklist [14] or the Adult Behavior Checklist [15], both with acceptable psychometric properties according to the manuals. The questionnaires assess competencies, strengths, adaptive functioning, and behavioral, emotional, and social problems and their scoring programs provide relevant multicultural norms [16]. The problem items are rated on a 3-point Likert scale (0–2; not true, somewhat true, or very true). Both questionnaires include Total, Internalizing, and Externalizing problem scores with a borderline clinical range from *T* = 60–63 and a clinical range above 63. The two questionnaires also include 8 syndrome scales truncated at the lower end of the scale (at *T* = 50) and with a borderline range from *T* = 65–69 and a clinical range of ≥70. Both questionnaires include subscales measuring anxiety and depression (anx/dep), withdrawn behavior, somatic symptoms, thought problems, attention problems, and aggression. Two subscales differ slightly: while the CBCL evaluates social problems, the ABCL evaluates intrusive behavior. The ASEBA questionnaires also provide a section for open-ended responses where parents/caregivers can describe strengths and concerns about their offspring.

#### Quality of life

Quality of life in affected and unaffected offspring was rated by the parents using the Inventory of Life Quality in Children and Adolescents (ILC) [17, 18]. A review of Norwegian ILC studies found satisfactory norms and measures of validity and reliability [19]. The ILC includes seven items on how well they cope at school and function when alone, with family members and other children, perceptions of their physical and psychological wellbeing, and finally an overall/global item. Each item is rated on a 1–5 scale (1 = very good through 3 = mixed, to 5 = very bad). The ILC Life Quality total score (LQ_0–28_) is calculated as a sum of the seven single items (reversed scores) and transformed so that the scores range from 0 - 28, where a value of zero indicates very low QoL and 28 very high QoL. A parent rated mean LQ_0–28_ score of 24.42 (SD = 3.15) has been reported for healthy Norwegian youth (*n* = 2563) [17]. For use with individuals affected by a health condition, the ILC includes separate impact questions related to how the medical condition and the medical controls affect the offspring and the parents, respectively, rated on a five-point scale from not at all burdened by [1] to very severely burdened by [5].

Parental quality of life was measured using the Satisfaction With Life Scale (SWLS) completed by both parents [20]. The five-item SWLS was developed to measure the cognitive component of subjective well-being. The items are: “In most ways, my life is close to my ideal”, “The condition of my life is excellent”, “I am satisfied with my life”, “So far I have gotten the important things I want in my life”, and “If I could live my life over, I would change almost nothing”. In the original validation study [20], the SWLS demonstrated an internal consistency of α = 0.87 and was found to be a valid and reliable measure of life satisfaction, suitable for use across a wide range of ages and applications [21, 22]. Each item is scored on a scale from 1 to 7 indicating very dissatisfied to very satisfied, with 4 as the neutral point. Scores ≥ 5 on the SWLS are considered to reflect the widely replicated finding of good quality of life in nonclinical samples, while 4 represents the neutral point [23]. We report a mean total score, calculated as the sum of all scores divided by the number of items. An overview of measures used in this study is presented in Table 1.

**Table 1:** Overview of measures used in the study for the different participants

Parent reported questionnaires	CLN3	siblings	parents
Hamburg Kohlschütter-scale for CLN3 [12]	x		
Child Behavior Checklist; CBCL [14] or Adult Behavior Checklist [15]	x	x	
Quality of life; ILC [18]	x	x	
Quality of life; SWLS scale [20]			x
Family background sociodemographic data			x

*Note:* ILC Inventory of Life Quality in Children and Adolescents, SWLS Satisfaction With Life Scale

#### Data analysis

Descriptive statistics, including means, standard deviations (SD), and ranges, are presented. Independent t-tests were utilized to examine mean score differences between individuals diagnosed with CLN3 disease and their sibling controls, as well as to assess any sex differences within these groups. Given that not all affected individuals had siblings in this already small sample size, independent t-tests were necessary to include all patients in the analysis. Because there was a dependency between the affected individuals and their siblings, paired sample t-tests were conducted to determine if this altered the results. The level of significance for all statistical tests was set at *p* < 0.05. Additionally, correlation analyses were performed to investigate associations between various variables. To quantify the magnitude of differences in means between groups, Hedges g’ was calculated using MedCalc for Windows, (Version 23.1.7; accessed February 14, 2025; MedCalc Software, Ostend, Belgium).

## Results

### Background characteristics

Data were collected on 20 individuals (13 males) with CLN3 disease, their parents and 16 siblings (9 males). There was no significant difference in the mean age of affected individuals, which was 15.2 years (SD = 8.6, range = 7–29, with five individuals > 18 years), compared to their siblings, whose mean age was 16.7 years (SD = 8.0, range = 7–29). There was no significant difference in the mean age of affected males and females (15.1, SD = 6.6, and 15.3 (SD 7.2), respectively. Six of the affected individual/sibling pairs were of opposite sex, eight had younger and eight had older healthy siblings. Males in the sibling group had were not significantly younger than females (14.4 versus 19.6 years, *t* = 1.28, df 14, *p* = 0.22).

The mean age at onset of CLN3 disease symptoms was 5.7 years (SD = 1.2, range = 4–8). The mean sum score on the Hamburg Kohlschütter scale was 7.80 (SD = 3.78, range = 1–14).

Sixteen of the 20 affected individuals lived with their biological parents, while the four eldest resided in community care. The mean age of the mothers was 47.2 years, while 49.6 years for fathers (SD = 7 for both), all but three couples were married. The parents were generally well educated, 50% had ≥ 16 years of education. Due to the care needs of their offspring, most parents did not work full-time; the mean work percentage was 43% for mothers and 72% for fathers, supplemented by a government-funded carer’s allowance.

Severity of CLN3 severity increased with chronological age, resulting in a significant positive correlation (*r* = 0.76, *p* < 0.001). Within the age groups, individuals aged 7–18 years had a Hamburg Kohlschütters Scale mean score of 9.1 (SD = 2.7) while individuals aged 18–29 years had a mean score of 3.6 (SD = 1.9) (*t* = −4.17, df = 18, *p* < 0.001).

There were no significant sex differences in severity of CLN3 disease (*t* = 0.56, df = 18, *p* = 0.58). However, as shown in Table 2, males consistently exhibited better mean scores.

**Table 2:** The CLN3 symptom severity evaluation; the Hamburg Kohlschütter-scale

Five areas and Sum	Mean (SD, range)		
	Males (n = 13)	Females (n = 7)	All (n = 20)
Vision	0.62 (0.77, 0–2)	0.57 (0.98, 0–2)	0.60 (0.82, 0–2)
Intellect	1.92 (0.64, 1–3)	1.57 (0.98, 0–3)	1.80 (0.77, 0–3)
Language	1.77 (0.83, 1–3)	1.57 (1.13, 0–3)	1.70 (0.92, 0–3)
Motor function	2.15 (0.80, 1–3)	1.71 (1.11, 0–3)	2.00 (0.92,0–3)
Epilepsy	1.85 (1.07, 0–3)	1.43 (1.51, 0–3)	1.70 (1.22, 0–3)
Total score	8.31 (2.90, 3–14)	6.86 (5.18, 0–14)	7.80 (3.78, 0–14)

*Note:* CLN3 ceroid lipofuscinosis neuronal 3; SD standard deviation

### Emotional and behavioral problems

Affected individuals scored within the borderline clinical range on the ASEBA total problem score and significantly higher than sibling controls, with mean scores of 63.1 (SD = 11.6) and 46.4 (SD = 11.4), respectively (*t* = 4.31, df = 34, *p* < 0.001). This difference represents a large effect size (g = 1.42).

Both the Internalizing (mean = 60.4, SD = 12.2) and Externalizing (mean = 57.8, SD = 11.6) problem scores were at the high end of the normal range but were significantly elevated compared to sibling controls (mean scores of 49.4 and 45.8, respectively, with Internalizing *t* = 2.91, df = 34, *p* = 0.006, Externalizing *t* = 3.33, df = 34, *p* = 0.002). The differences in mean Internalizing and Externalizing scores between groups represent large effect sizes (g = 0.90 and 1.01, respectively).

Detailed analyses showed that affected individuals aged 7–18 years scored in the clinical range on the CBCL total problem scale, with both the internalizing and externalizing problem scales falling within the clinical and borderline ranges, significantly higher than sibling controls (Table 3). Individuals aged 19–29 years scored within the normal range on the three ABCL problem scales but still demonstrated significant differences from sibling controls on the Total and Externalizing scales (Table 3).

**Table 3:** Mean problem scores on the two ASEBA Behavior checklists for affected individuals and sibling controls

ASEBA Problem scores	CLN3 mean (SD)	Siblings mean (SD)	Statistics
**CBCL** Total problems	66.3 (SD 9.9)	49.7 (SD 13.5)	t = 3.48, df 22, *p* = 0.001, g = 1.39
Internalizing	64 (SD 10.4)	52.8 (SD 11.7)	t = 2.45, df 22, *p* = 0.023, g = 1.40
Externalizing	60.5 (SD 11.6)	48.4 (SD 12.7)	t = 2.42, df 22, *p* = 0.024, g = 0.98
**ABCL** Total problems	57.2 (SD 5.0)	42.3 (SD 6.9)	t = 4.01, df 10, *p* = 0.002, g = 2.46
Internalizing	52.4 (SD 9.3)	45.1 (SD 6.4)	t = 1.61, df 10, *p* = 0.14, g = 0.88
Externalizing	52.0 (SD 5.8)	42.6 (SD 5.0)	t = 3.01, df 10, *p* = 0.013, g = 1.68

*Note:* CLN3 ceroid lipofuscinosis neuronal 3; CBCL Child Behavior Checklist

ABCL Adult Behavior Checklist; SD standard deviation

Within the CLN3 group, there were no significant mean sex differences on the ASEBA Total problem score (males (65.1, SD = 11.0) and females (59.4, SD = 12.5) (*t* = 1.04, df = 18, *p* = 0.31). The same lack of sex differences was found for siblings (males (45.7, SD = 13.1) and females (47.3, SD = 10.0) (*t* = 0.27, df = 14, *p* = 0.79).

**Fig. 1:**
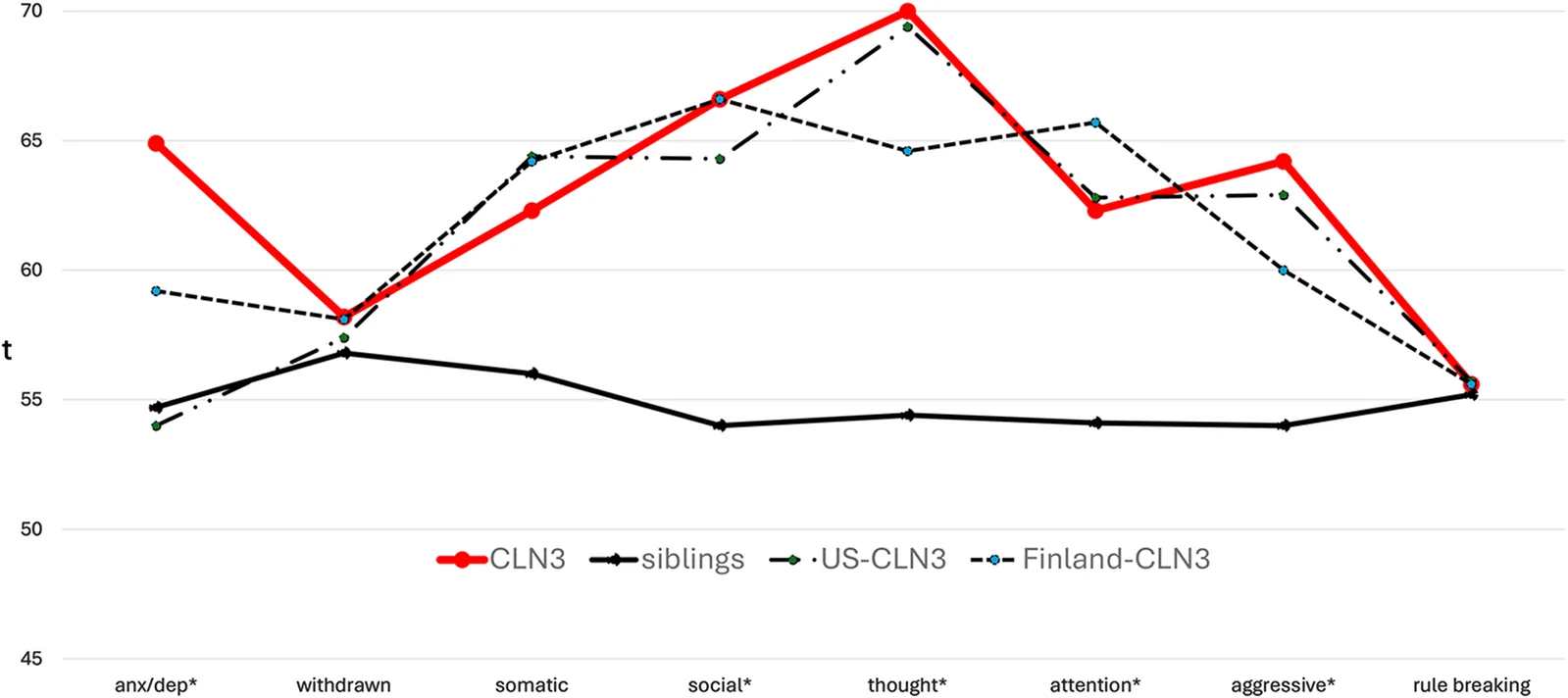
The CBCL syndrome scales in CLN3 disease and sibling controls, and CLN3 data from Finland and Usa

**Fig. 2:**
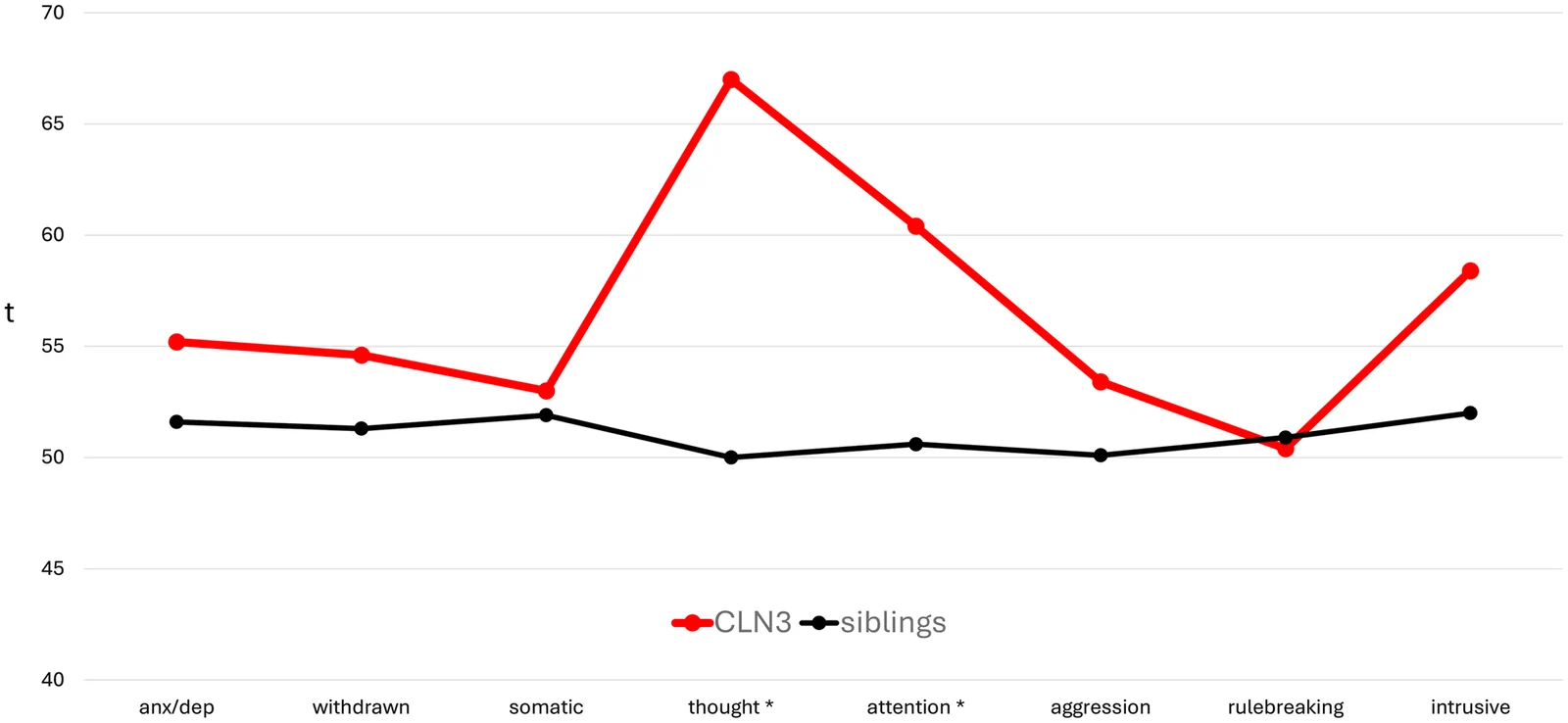
The ABCL syndrome scales in CLN3 disease and sibling controls

The syndrome scores are presented for the CBCL and ABCL are presented in Figs. 1 and 2, respectively, with significant group differences marked with asterisks. For the CBCL, all subscales except “withdrawn”, “somatic” and “rule breaking” were significantly higher in affected individuals compared to their siblings, indicating a pronounced difference in the younger patient group. In contrast, for the ABCL, which is used for the age group > 18 years, only the “thought problems” and “attention” subscales were significantly higher in affected individuals compared to sibling controls.

The majority of parents used the free description section to articulate strengths and concerns. They frequently identified a sense of humor (9/20), grit, and determination (10/20) as key characteristics of the affected individuals, while empathy (10/16) and dutifulness (7/16) were commonly attributed to sibling controls. Parents’ concerns primarily revolved around the challenges associated with having CLN3 disease (11/20), as well as the impact of being a sibling to someone affected by this condition (3/16).

Several CBCL and ABCL single items were identified as significant challenges for affected individuals. The items “demands much attention”, “has trouble sleeping”, “speech problems” and “can’t concentrate” were found to be common difficulties across age groups (See supplement 1 for a list of the ten most frequently mentioned challenges on CBCL and ABCL).

### Quality of life

Mothers (*n* = 20) and fathers (*n* = 19) rated their own quality of life as low, with scores of 3.41 and 3.80, respectively, both falling below the neutral point [4] on the SWLS.

For the affected individuals, the mean ILC total score (LQ_0–28_) reported by parents was 14.80 (SD = 5.40), compared to 24.88 (SD = 3.40) for siblings, indicating a significant difference between the two groups (*t* = −6.50, df = 34, *p* < 0.001), with a very large effect size (g = 2.13).

**Fig. 3:**
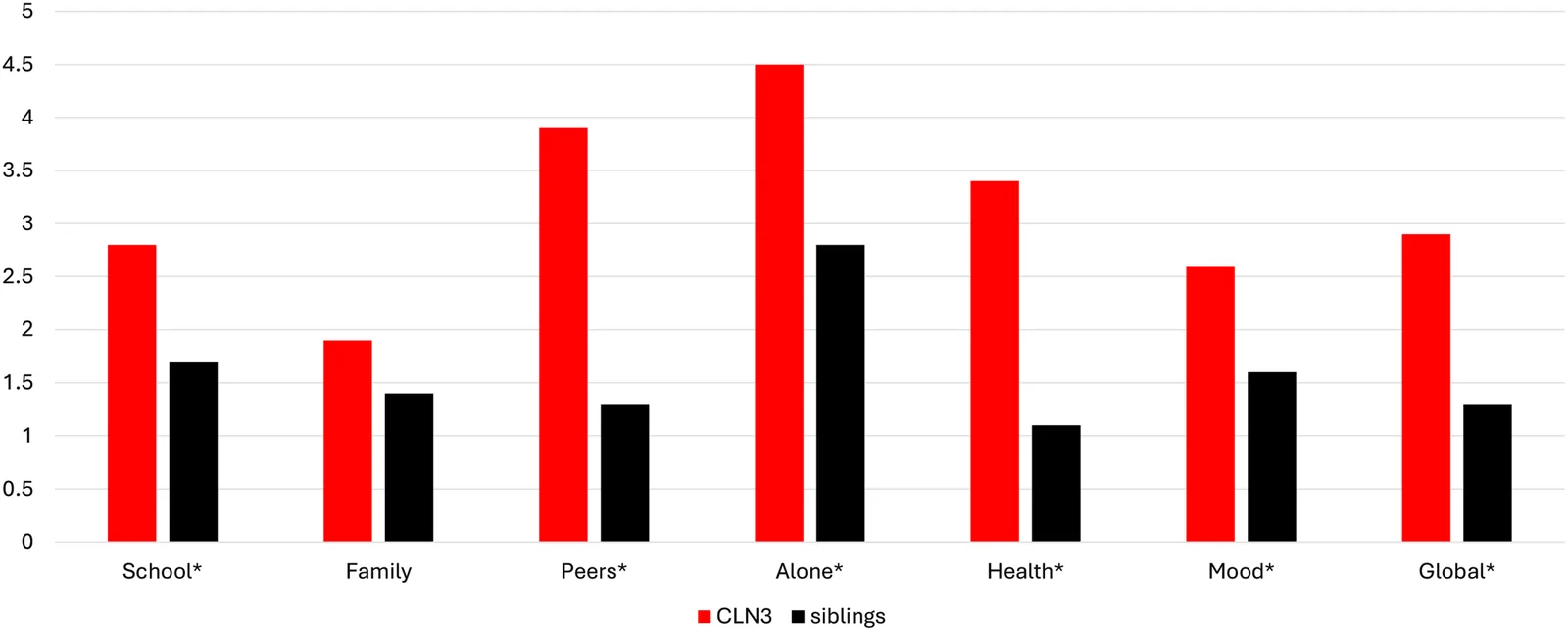
ILC subscales in CLN3 disease and sibling controls

Apart from the Family subscore on the ILC, all other subscores were significantly worse (all *p*’s < 0.005) among affected individuals compared to sibling controls (Fig. 3).

The five individuals with CLN3 disease aged 19–29 years had a lower mean LQ_0–28_ compared to those aged 7–18 years, with scores of 10.20 (SD 4.0) versus 16.33 (SD 5.0), respectively. This difference was statistically significant (*p* = 0.01) with a large effect size (g = 1.28). No significant age effects were observed in the sibling group (*r* = 0.17). Additionally, there were no significant sex differences in the mean ILC total score among affected individuals (males: 14.61 (SD = 5.45) and females: 15.14 (SD = 5.70), *t* = −0.20, df = 18, *p* = 0.84).

The two ILC impact questions revealed that both the affected individual and the parents were severely burdened by CLN3 disease (means = 4.1 and 4.0), but only somewhat burdened by the medical controls (means = 2.9 and 3.2).

While there were significant associations between the severity of CLN3 disease symptoms and measures of QoL within the family, this was not the case with the ASEBA total problem score (Table 4). Also noteworthy was the significant association between the ASEBA problem score and the QoL score observed only in siblings.

**Table 4:** Correlations between severity of CLN3, the ASEBA total problem score and measures of quality of life

	CLN3 severity	ASEBA Total	QoL in CLN3	QoL, mother	QoL, father	QoL, siblings
Severity of CLN3	x	0.17	0.78**	0.31	0.54*	-
ASEBA-total		x	0.16	−0.06	−0.18	0.75**
QoL in CLN3			x	0.46*	0.60**	0.26
QoL, mother				x	0.76**	−0.05
QoL, fathers					x	0.26
QoL, siblings						x

*Note:* CLN3 ceroid lipofuscinosis neuronal 3, severity was measured by the Hamburg Kohlschütters Scale

QoL Quality of life; ** significant at the 0.01 level, * significant at the.05 level

In all group comparisons above, paired sample t-tests conducted with affected individuals and their sibling controls yielded *p*-values similar to those obtained in independent t-tests, indicating only marginal differences.

## Discussion

In this national sample, we found that individuals with CLN3 Batten disease exhibited significantly more emotional and behavioral symptoms and lower quality of life compared to their siblings. This was perhaps not surprising due to CLN3 disease being a neurodegenerative disorder, and groups of individuals with neurological disorders are generally known to experience higher rates of comorbid emotional disorders [24, 25].

### Emotional and behavioral problems

The significantly higher scores in affected individuals compared to their siblings underscore the specific impact of CLN3 disease on emotional and behavioral symptoms. This confirms clinical observations and aligns with findings from prior research in Finland [2] and the USA [3]. Our detailed analysis stratified by age groups demonstrated that individuals aged 7–18 years exhibited more pronounced emotional and behavioral symptoms compared to those aged 19–29 years. This finding suggests that younger affected individuals may require more attentive monitoring and intervention of these symptoms. This contrasts with the US study from 2006 which found no significant correlation between chronological age and the CBCL total problem score [3]. The discrepancy may be attributed to our inclusion of individuals over 18 years of age, which provides a broader age range than that examined in the US study; but similar to a subsequent larger study with children and adults using the CBCL identifying an inversed U-curve [4].

The elevation in internalizing and externalizing scores suggests that affected individuals experience both inward-focused emotions like anxiety and depression, as well as outward-focused behaviors such as aggression. However, for the CBCL (used for the younger age group), only two subscales did not show significant differences between affected individuals and their siblings. The absence of group differences on the ‘withdrawn’ subscale may cohere with parents describing key characteristics such as “grit”, “determination”, and a “sense of humor” in their affected offspring in the open-ended responses on the CBCL. The finding that there were no group differences on the “somatic” subscale could be somewhat surprising, considering the medical condition of the affected individuals. However, the single items on this scale primarily refer to aches, nausea, and skin problems without known medical cause and are generally associated with somatization disorders.

We did not observe any significant sex difference in emotional and behavioral symptoms within the CLN3 group. This finding contrasts with the previous Finnish study that highlighted sex differences in symptom expression at a mean age of 15 years [2], with females exhibiting higher levels of anxiety and depression. However, our results are consistent with the US study that did not identify significant sex differences at a mean age of 12 years, even though the mean age in our study closely matched that of the Finnish study.ackma

The absence of significant sex differences may indicate that they do not exist, although the small sample sizes in CLN3 studies across all three countries make such differences challenging to detect.

### Quality of life

The present study found a stark contrast in QoL between affected individuals and their siblings, in line with previous study findings that CLN3 disease significantly burdens those afflicted [5, 6]. Notably, the sibling mean QoL scores align closely with those reported for Norwegian youth, indicating that the siblings in the present study have good QoL despite the strenuous circumstances. This contrasts with Fullerton et al. (2017), which suggested that siblings of children with life-limiting conditions face a reduced QoL, although the findings have been mixed across different chronic illnesses [9]. It is noteworthy that we only had parent reports in the present study, and parents may perceive siblings’ situations more positively than they would themselves.

Though the present study found no significant sex differences in ILC scores among affected individuals, this contrasts with a previous study reporting that females experienced earlier physical decline, and lower health-related quality of life specifically in the domain of physical function [5]. This discrepancy may reflect the use of different rating instruments and our relatively small sample size, which reduce power to detect sex differences. Additionally, the present sample included more males than females, further limiting power. A recent review summarizes the existing literature on sex differences in CLN3 and concludes that results are inconsistent and that further study is warranted [26].

We found a reduced QoL among the parents in our study, consistent with a previous study using qualitative interviews [1]. This underscores the need for family support systems and interventions. The data revealed that the majority of parents were not engaged in full-time employment, with mothers working an average of 43% and fathers 72%, highlighting a significant caregiving burden. This is considerably lower than the mean rate of employment for parents in the relevant age group (40–54 years) which is 80% for mothers and 85% for fathers, according to Statistics Norway.

Although the families received government-funded carer’s allowances, the reduction in work hours likely resulted in economic repercussions, as well, in line with a previous qualitative study [1].

Four out of the five eldest individuals with CLN3 disease resided in community care facilities, a group with lower QoL scores compared to the younger group. The age-related decline associated with CLN3 disease necessitates tailored care approaches as individuals grow older, including round-the-clock support provided by staff providing medical care to manage the disease.

The lack of association between ASEBA scores and familial QoL suggests that other factors, such as the consequences of CLN3 disease itself, may have a more substantial influence on QoL than emotional and behavioral symptom severity alone. The responses to the two impact questions related to QoL suggested that medical controls were manageable for both the affected individual and the parents, while the severe burden of CLN3 disease itself required enhanced support.

### Strengths and limitations of the study

This study had several strengths, including a national sample and comprehensive measures of emotional and behavioral symptoms alongside quality-of-life assessments for the affected individuals, parents and siblings. However, important limitations must be acknowledged. Firstly, the rarity of CLN3 disease resulted in a small sample size, limiting the statistical power and potentially affecting the robustness of the findings. Although a national sample was used, it constrains the generalizability of the results to other cultures, emphasizing the need for multisite studies across borders. Such studies are necessary for validating findings, particularly concerning subgroup differences, such as those related to sex. Because CLN3 is an ultra‑rare disorder, larger multisite and international studies are likely required both to increase sample size and to assess cross‑country differences that could arise from variation in healthcare systems, social services, genotype distributions, or cultural and language effects on rating instruments. We therefore recommend and highlight the need for coordinated international efforts.

Moreover, the cognitive decline associated with CLN3 disease may have posed challenges regarding the interpretation and response to questionnaire items. Parents may have found it difficult to accurately relate to or understand questions, considering their offspring’s cognitive challenges. In the older age group, expression of emotional and behavioral symptoms may be constrained by diminishing physical capabilities. This pattern is analogous to findings in the dementia literature, where positive behavioral symptoms often follow an inverted‑U course and later give way to negative symptoms such as apathy [27]. Because our study is cross‑sectional, we cannot track within‑patient transitions from positive to negative symptomatology or determine whether lower reported symptom levels in more severely affected individuals reflect true improvement versus reduced ability to communicate distress. Specifically, the observed lower scores of emotional and behavioral symptoms in adults with CLN3 disease may also be attributed to the limitations of the ABCL, although they corroborate findings from the previously mentioned US study from 2013 using the CBCL in both children and adults with CLN3 disease [4].

Additionally, the lack of self-reports from affected individuals and siblings restricts insights from direct perspectives. Finally, the cross-sectional design makes it difficult to draw conclusions beyond this single time point. Overall, these limitations underscore the need for future research to employ larger, more diverse samples and to include longitudinal follow-up.

## Conclusions

We found higher occurrence rates of emotional and behavioral symptoms among individuals with CLN3 disease compared to their siblings. Still, the scarcity of quantitative research focusing on these symptoms and QoL in affected individuals is still apparent. This gap underscores the need for multicentre trials and longitudinal studies to enhance our understanding and devise effective interventions for these symptoms. A sibling study with self-reports is warranted, as it would allow siblings to contribute firsthand information necessary for improved understanding of family dynamics.

### Clinical implications

This study offers valuable insights for clinicians and healthcare providers, emphasizing the importance of incorporating integrated psychiatric evaluations into the standard care practice for individuals with CLN3 disease. Recognizing the impact of family strain on the QoL of both parents and siblings is essential to providing adequate support for the entire family.

## Data Availability

The participants of this study did not give written consent for their data to be shared publicly, so due to the sensitive nature of the research, supporting data are not available.
